# Sparse Spectro-Temporal Receptive Fields Based on Multi-Unit and High-Gamma Responses in Human Auditory Cortex

**DOI:** 10.1371/journal.pone.0137915

**Published:** 2015-09-14

**Authors:** Rick L. Jenison, Richard A. Reale, Amanda L. Armstrong, Hiroyuki Oya, Hiroto Kawasaki, Matthew A. Howard

**Affiliations:** 1 Department of Psychology, University of Wisconsin Madison, Madison, Wisconsin, United States of America; 2 Department of Neurosurgery, University of Iowa, Iowa City, Iowa, United States of America; University of Salamanca- Institute for Neuroscience of Castille and Leon and Medical School, SPAIN

## Abstract

Spectro-Temporal Receptive Fields (STRFs) were estimated from both multi-unit sorted clusters and high-gamma power responses in human auditory cortex. Intracranial electrophysiological recordings were used to measure responses to a random chord sequence of Gammatone stimuli. Traditional methods for estimating STRFs from single-unit recordings, such as spike-triggered-averages, tend to be noisy and are less robust to other response signals such as local field potentials. We present an extension to recently advanced methods for estimating STRFs from generalized linear models (GLM). A new variant of regression using regularization that penalizes non-zero coefficients is described, which results in a sparse solution. The frequency-time structure of the STRF tends toward grouping in different areas of frequency-time and we demonstrate that group sparsity-inducing penalties applied to GLM estimates of STRFs reduces the background noise while preserving the complex internal structure. The contribution of local spiking activity to the high-gamma power signal was factored out of the STRF using the GLM method, and this contribution was significant in 85 percent of the cases. Although the GLM methods have been used to estimate STRFs in animals, this study examines the detailed structure directly from auditory cortex in the awake human brain. We used this approach to identify an abrupt change in the best frequency of estimated STRFs along posteromedial-to-anterolateral recording locations along the long axis of Heschl’s gyrus. This change correlates well with a proposed transition from core to non-core auditory fields previously identified using the temporal response properties of Heschl’s gyrus recordings elicited by click-train stimuli.

## Introduction

**Fig 1:**
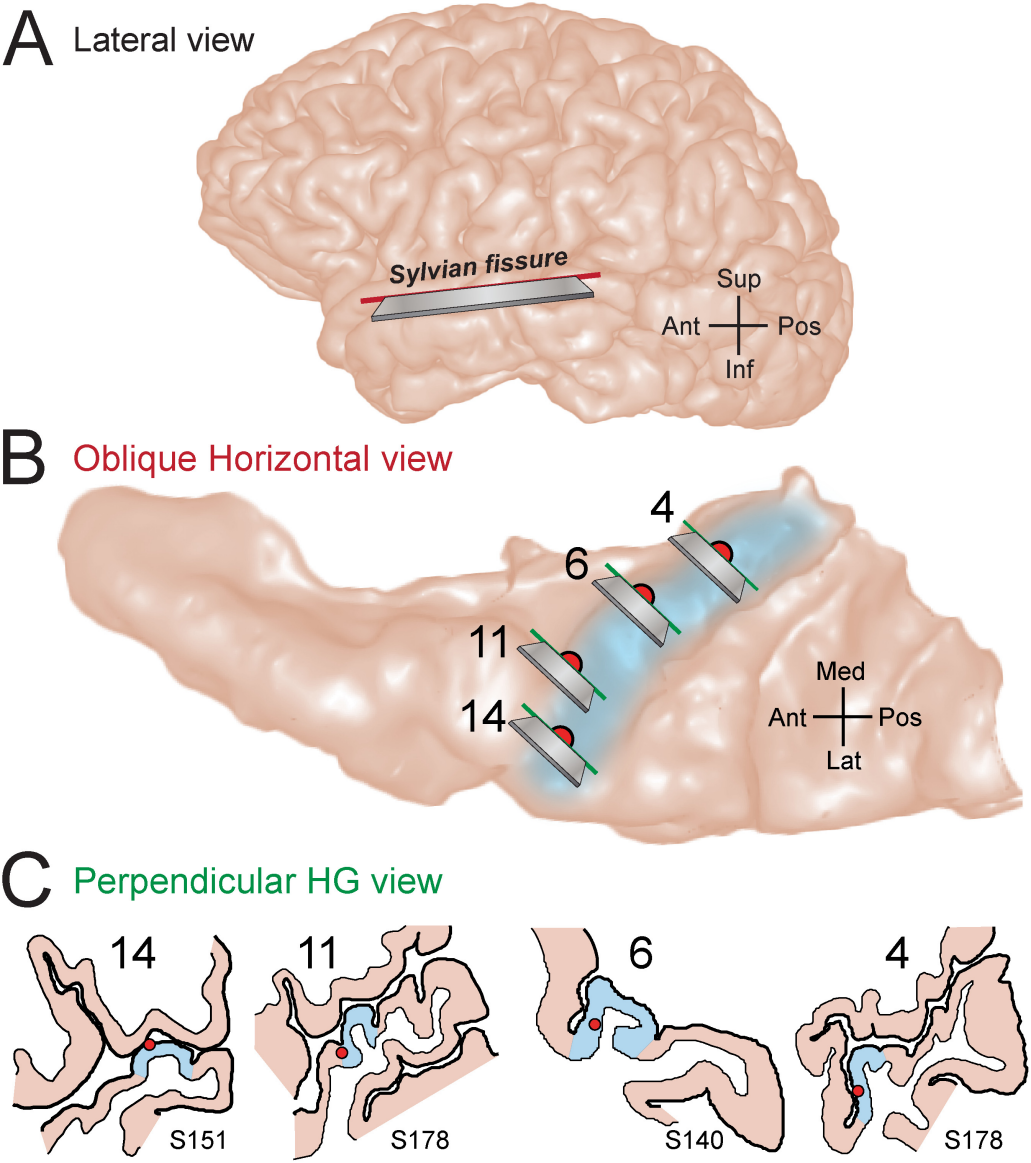
Locations of electrode recording sites within the superior temporal plane. **(A**) MRI lateral-view rendering of a typical human left hemisphere. The Sylvian fissure is not visible from the cortical surface. The superior temporal plane was revealed along a section oriented at an oblique horizontal plane (solid red line with razor blade inset). (**B**) MRI rendering of superior temporal plane viewed from superior aspect. Light blue shading denotes the location of the obliquely oriented Heschl’s gyrus. The estimated locations of four recording sites selected from three different subjects (S140, S151, and S178) were projected to the surface of this illustrative brain and marked with filled red circles. MRI cross-sectional images containing the recording sites were obtained from sections oriented at an oblique frontal plane (solid green lines with razor blade inset), approximately perpendicular to the long axis of Heschl’s gyrus. (**C**) Line drawings of MRI cross sections show the position of the recording sites within the grey matter of Heschl’s gyrus for individual subjects.

Human auditory cortex is composed of multiple fields distributed both on the exposed lateral surface of the superior temporal gyrus (Fig 1A) and in areas on the supratemporal plane buried within the Sylvian fissure (for review see: [1–4]). The hidden supratemporal plane can be visualized on the surface that results from an oblique horizontal sectioning, perpendicular to the lateral hemisphere (Fig 1B). Chronically implanted electrodes in human subjects [5] have been used to identify a portion of Heschl’s gyrus (Fig 1B and 1C) on the supratemporal plane that is consistent with it being the primary and primary-like (core) auditory cortex [6–9].

Responses recorded simultaneously from Heschl’s gyrus and other auditory responsive cortical sites differ in a number of response criteria; including sensitivity to general anesthesia, phase-locking capacity, response latency, and spectral tuning (for review see: [3, 10, 11].) Neural spectral tuning has been considered one mechanism by which both communication and non-communication sounds are discriminated, and auditory cortex has been considered a place where the requisite neurons are located. Single neurons in Heschl’s gyrus of human subjects recorded with implanted electrodes have been found that are extraordinarily narrowly tuned (“ultra sharp”), and their frequency selectivity may account for a listeners threshold of frequency discrimination as measured psychophysically [12]. Frequency tuning curves similar to those recorded in auditory cortex of laboratory animals have also been recorded in human Heschl’s gyrus, and their distribution has confirmed the presence of at least one tonotopic field in Heschl’s gyrus core [13]. Estimates of spectral tuning are commonly obtained with stimulus sets composed of pure tones or complex sounds (e.g. narrow-band noise). In animal studies, features derived from spectral tuning estimates have proven of great value in describing functional organizations, patterns of connectivity, sound localization behaviors, and communication sound processing (for review see: [14]). As such, improvements in such estimates continue to be of importance to neurophysiologic studies. Here, we present a contemporary approach to estimating frequency and temporal tuning, which addresses low signal-to-noise data that is commonly recorded from human Heschl’s gyrus.

Spectro-Temporal Receptive Field (STRF) derivations represent an advancement in characterizing both frequency and temporal tuning. STRFs of single neurons have traditionally been derived by estimating a transfer-function between a white-noise input stimulus and resulting neural action potentials. This approach is generally known as a spike-triggered average (STA) or reverse-correlation. Reverse-correlation was originally developed to estimate filter characteristics of auditory peripheral afferents [15], although continued advances made in the stimulus structure have provided STRF estimates at many sites along the central auditory pathway including neocortex [16–21]. Natural stimuli have also been used to derive STRFs, which required a normalization to accommodate the statistically non-white auditory stimulus [22]. This was accomplished by scaling the STA by the inverse of the stimulus autocorrelation matrix. In general, STA-based models require a large amount of data to average out the noise (i.e. non-stimulus related responses). More recently, the parametric Generalized Linear Model (GLM) was used to estimate STRFs based on point-process encoding models [23–25]. Given that the number of covariates (i.e. predictors) is typically large, most GLM parameter estimates require some form of regularization or sparse constraint that tends to drive the covariate coefficients to zero. A large amount of multi-disciplinary research has gone into the development of sparse models [26]. The basic idea is to represent the data with a transformation using as few variables as necessary, and thereby, as parsimoniously as possible.

The frequency-time structure of the STRF tends toward grouping or non-uniformity in different areas of frequency-time combinations. One approach to preserving this structure has been to formulate Bayesian priors for locality and smoothness as shown by Park and Pillow [25]. The locality constraint is the most similar and relevant to the approach used in the present study. Although this approach encourages the emergence of local filters, it would tend to discourage the emergence of multimodal filters, unless the modes are truly periodic in frequency in time (locality in Fourier domain). Here we present an alternative approach that extends sparse GLM from action on single covariates to groups of covariates that encourage local filtering in frequency and time [27]. We demonstrate a form of group sparsity applied to GLM estimates of STRFs that reduces noise while maintaining the structure within well-circumscribed boundaries. Although the GLM methods have been used to estimate STRFs in various species, this study is the first to apply these techniques to intracranial recordings in the awake human brain. We found a mixture of narrow, broad, and complex spectral and temporal tuning in Heschl’s gyrus, that appears to be in contrast to an earlier finding of predominantly narrow tuning in human Heschl’s gyrus [12].

A number of studies have now shown that spiking activity often covaries with LFP power in the high-gamma (High-*γ*) range of frequencies (70–200 Hz) [28–31]. High-*γ* power changes have been observed in human electrocortiograms (ECoG) under a wide variety of behaviors, including auditory and speech related tasks [32–37]. Here we are interested in how well the GLM derived STRF from High-*γ* power compares to that of the STRF based on spiking activity. In contrast to the STA or cross-correlation methods, the GLM also allows for the introduction of covariates related to the history of neural activity, and therefore factors-out the intrinsic activity from that of the extrinsic stimulus. We found that in the majority of recording sites, multi-unit firing-rate history contributed significantly to the observed High-*γ* power during STRF estimation, which suggests an association between these two signals.

## Materials and Methods

### Ethics Statement

Human subjects in this study were neurosurgical patient volunteers that had medically refractory epilepsy and were undergoing chronic invasive ECoG monitoring to identify potentially resectable seizure foci. All subjects were native English speakers and underwent audiometric evaluation and none were found to have a hearing deficit. Intracranial recordings revealed that the auditory cortical areas on the superior temporal plane were not epileptic foci. The University of Iowa and University of Wisconsin-Madison Institutional Review Boards (IRB) approved the study over the course of data collection and analysis. Informed consent was obtained from each patient after the nature and possible consequences of the studies were explained to them. Patients provided their written informed consent to participate in this study. The original IRB approved, signed Informed Consent Document was placed in our research files. A copy of the signed Informed Consent Document was given to the patient, and a copy of the signed Record of Consent form was placed in the patient’s electronic medical record. Patients did not incur additional risks by participating in this study. The decision to implant the electrodes, as well as their location, was driven solely by medical considerations.

### Neurophysiological Recordings

Details of electrode implantation and data collection have been described previously [9, 11, 38]. In neurosurgical patients undergoing chronic electrophysiological monitoring for medically refractory epilepsy, a multi-contact electrode can be placed along the long axis of Heschl’s gyrus to allow clinical electrocorticographic (ECoG) monitoring of the dorsal surface of the temporal lobe. When hybrid clinical-research depth electrodes are placed within the gyrus it is also feasible to obtain microelectrode recordings from multiple locations along Heschl’s gyrus. Simultaneous ECoG recordings were obtained from 14 microwire contacts distributed along the length of a hybrid-depth electrode, stereotactically implanted into Heschl’s gyrus, and roughly parallel to its long axis [13, 39]. ECoG signals were amplified, filtered (2.2–7500 Hz, 3dB-corners, 6 dB/octave rolloff), digitized at a sampling rate of 12,207 Samples/sec, and stored for subsequent offline processing that included action potential sorting [40, 41] and extraction of continuous High-*γ* band power. The hybrid-depth electrode remained in place up to two weeks during continuous clinical ECoG monitoring. Experiments were carried out in a dedicated electrically-shielded suite in The University of Iowa General Clinical Research Center. The room was quiet, with lights dimmed. Subjects were awake and reclining in a hospital bed or an armchair. Stimuli were presented in a passive listening paradigm.

### Anatomical Localization

Reconstruction of the anatomical locations of the microwire recording sites in the superior temporal plane, together with their detailed positions within the cortical gray matter, was performed using software developed in house [8, 9, 42]. The surface of the hidden supratemporal plane can be visualized by digitally removing the frontal and parietal lobe brain regions overlying the fissure. This provides a surface view of Heschl’s gyrus which is oriented obliquely within the supratemporal plane, with the most superficial portion terminating in the anterior portion of lateral superior temporal gyrus. In brief, recording site locations were first manually identified using post-implantation magnetic resonance (MR) images, and subsequently mapped onto preoperative MR scans using non-linear warping (Catmull-Rom spline interpolant). Both the trajectory of the depth electrode and the locations of microwire recording sites were then projected onto an MR rendering of the superior temporal plane as viewed within the Sylvian fissure (Fig 1B). Serial MR cross-sectional images (oblique coronal plane, perpendicular to the long axis of Heschl’s gyrus) containing each of the recording sites were also obtained and outline drawings of these sections indicated each contact’s location within the cortical depth (Fig 1C).

### Stimulus Generation and Delivery

The term gammatone was introduced by Aertson and Johannasma [43] originally inspired by vocalizations of the grassfrog. A similar function was used to parametrically describe the impulse response of the auditory nerve single-units in cat [44]. Patterson and colleagues went on to demonstrate that the function was representative of psychophysical auditory filters [45]. A gammatone filter is defined as

$$g\left(t\right)=\frac{a\;t^{n-1}\text{cos}\left(2\pi F_{c}t+\varphi \right)}{e^{2\pi \;bw\;t}}$$ (1)

where *t* is time, *a* = 1 is gain, *n* = 1 is the filter order, *F*_*c*_ is the center frequency, *φ* is phase.

The bandwidth (bw) was scaled by 1.019 of the equivalent rectangular bandwidth (ERB: an approximation to the bandwidths of the filters in human hearing at each point along the cochlea) defined as

$$ERB={\left[{\left(\frac{F_{c}}{Q_{ear}}\right)}^{n}+bw_{\text{min}}^{n}\right]}^{\frac{1}{n}}.$$ (2)

The ERB parameters were based on recommendations for human auditory filters *Q*_*ear*_ = 9.26447;*bw*_min_ = 24.7 by Glasberg and Moore [46]. Gammatone stimuli have been used previously for estimating spectral receptive fields [21, 47].

**Fig 2:**
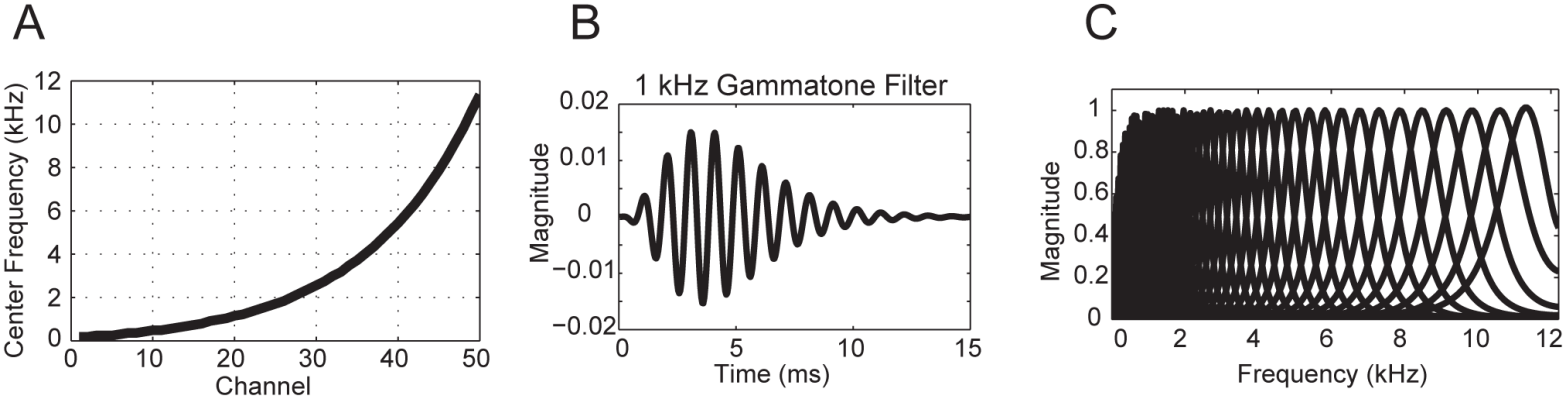
Gammatone stimuli. **A**) Human cochleotopic map of center frequencies from 100 to 11,234 Hz. **B**) Gammatone signal at 1 kHz center frequency. **C**) Gammatone filter bank with 50 channels.

Our implementation of an auditory filter-bank (Fig 2) was based on a the human cochleotopic map derived by Slaney [48] where each center frequency (Fig 2A) was computed by

$$\begin{array}{ll} Fc\left(n_{Fc}\right) & =-Q_{ear}\;bw_{\text{min}}+\;\left(F_{high}+Q_{ear}\;bw_{\text{min}}\right)\\ & \times \text{exp}\left\{n_{Fc}\;\left(\text{log}\left(F_{low}+Q_{ear}\;bw_{\text{min}}\right)-\text{log}\left(F_{high}+Q_{ear}\;bw_{\text{min}}\right)\right)/N_{frequency}\right\} \end{array}$$ (3)

and *n*_*Fc*_ is the Fc index, *N*_*filter*_ is the total number of filters, *F*_*low*_ is the lowest and *F*_*high*_ is the highest frequency (Nyquist) in the filter bank.

The STRF can be viewed as a definition of the stimulus-response characteristics of a neuron, and specifically as a linear filter impulse response that encompasses each frequency channel of interest. The goal then is to mathematically characterize estimates of the coefficients of a bank of filter channels. In this case, STRFs were obtained by randomly presenting gammatone blips, equally spaced on the cochleotopic scale noted above. Here, tone blips for each of *N*_*frequency*_ = 50 frequencies were presented according to a Bernoulli process x_*f*_∼*B*(1,*p*) with p = .02 for each blip onset (i.e., each 50 ms interval). Given the small p, the process could also be considered Poisson. The expected value of the number of gammatone blips across all frequencies was a rate of 20/s. The length of the impulse response for each frequency blip was 2 seconds (T = 40). The stimulus-state matrix *x*_*f*_(*t*) therefore determined whether a particular gammatone blip was turned on or off at any given 50 ms interval over a total of 6000 intervals or 5 minutes. The acoustic stimulus *y*(*t*) was generated using Gaussian noise excited gammatone blips in each frequency channel, and then aggregated across channels.

The stimuli were delivered to both ears via insert earphones (ER4B, Etymotic Research, Elk Grove Village, IL) that were integrated into custom-fit earmolds and presented at a comfortable level, about 30–50 dB above hearing threshold. Stimulus delivery and data acquisition were controlled by a TDT RP2.1 and RX5 or RZ2 real-time processor (Tucker-Davis Technologies, Alachua, FL).

### ECoG Processing

Prior to offline processing, ECoG data was pre-processed to remove large-amplitude time-transients, followed by removal of line noise according to an adaptive-filter procedure. Time-transients were identified by iteratively applying the z-score transform to raw data, at each step discarding values exceeding 10 until no further values were discarded. The discarded signal at removed transients was smoothed with a hamming window. Line noise suppression applied a threshold to the coefficients of a time-frequency decomposition, discarding those above the threshold and calculating the noise-filtered signal through the inverse transform. The time-frequency decomposition employed a frequency-domain implementation of complex demodulation [49], chosen for its efficiency and minimal susceptibility to spectral leakage artifacts as compared to alternative decompositions that rely on moving time windows (details to be provided in Kovach, submitted). In brief, line noise peaks in the spectrum were identified using overlapping 0.25Hz bands and time averaging. The average was log transformed and fitted with an 8^th^ order polynomial, giving a baseline power at each frequency. Line-noise contaminated baseline values were discarded after normalization amplitude and kurtosis thresholding.

#### Sorting single and multi-unit activity

Action potentials (spikes) for single neurons (units) or multi-units were identified using the continuous high band-width, high-impedance recordings from microwire contacts sampled at 12,207 Samples/sec. The impedance of microwire electrode sites was obtained in only one subject, because University of Iowa hospital medical engineering only permitted that measurement in this single case. The in-vivo impedance for the 14 contacts in this single subject ranged from 100 to 900 Kohm.

**Fig 3:**
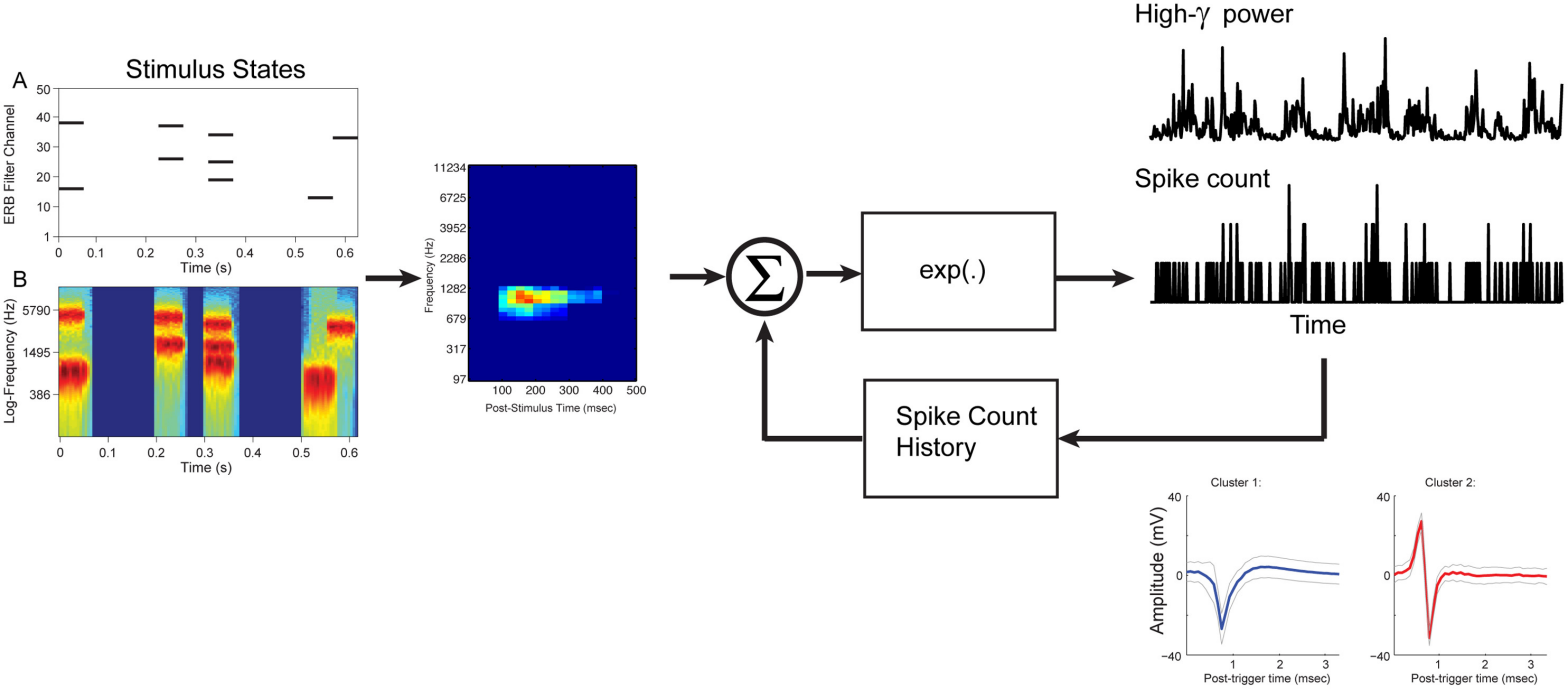
Generalized Linear Model (GLM) schematic. Stimulus-state matrix (A) is convolved with the Poisson estimated STRF, and exponentially transformed to generate a Poisson counting process. The corresponding stimulus Log-Spectrogram shown in lower panel (B). Alternatively, the stimulus-state matrix is convolved with the Log-normal estimated STRF and generates an exponential transformed High-*γ* process. Either model can include feedback of the spike-count history. Representative sorted multi-unit clusters from the same LFP recording are shown in the bottom right insets.

Candidate spikes were selected by amplitude thresholding, decomposed using wavelet analysis, and clustered using the algorithm developed by Quiroga et al. [40, 41]. Detailed descriptions of the methods used in his algorithm were presented in these and earlier manuscripts [50]. Briefly, an empirical voltage threshold was employed initially to identify potential candidate spikes, followed subsequently by a clustering of similar waveforms that depended on a single GUI controlled parameter (so-called paramagnetic temperature). At low temperatures, all candidate spikes will be considered as a single cluster, alternatively at high temperatures, the data are partitioned into several clusters with only similar candidate spikes in each. In this unsupervised clustering step, a wavelet analysis based on a Haar wavelet was employed to decompose candidate spikes into a nine-dimensional feature space. In our implementation, spikes were initially clustered with similar shapes to the same cluster using this unsupervised stochastic nonparametric superparamagnetic approach [50]. This automatic procedure was followed by an iterative manual adjustment of the threshold and temperature in order to empirically maximize the peak-to-peak amplitude of a single cluster that was used for further analyses. Generally, these single clusters with the largest peak-to-peak amplitude often exhibited a focused STRF where the discharge rate outside the focal area was minimal or zero. In other clusters, however, this was not the case as evidenced by substantial spontaneous discharges and/or the presence of foci where the relative discharge rate was suppressed. Rarely, a 2^nd^ cluster was identified by a substantially larger peak-peak amplitude and shape. Our iterative manual procedure resulted in 139 multi-unit clusters together with only 14 cases of a 2^nd^ single-unit cluster. Examples are shown in Fig 3. Spike-counts were obtained by binning spike times into 25 ms intervals.

#### High-*γ* band extraction from local field potential

The sample of ECoG recorded from a single electrode contact is often termed the local field potential (LFP) since the neural sources contributing to the potential have a restricted spatial extent [51]. LFP power in the High-*γ* range of frequencies has been hypothesized to represent the average firing of neurons near the recording site but weighted according to their distance from the electrode [28–31]. To extract a High-*γ* band signal from the LFP, the latter was down-sampled to 400 Samples/sec, and then filtered using a finite-impulse-response filter, band-passed between 70 and 150 Hz, and applied in the forward and reverse directions. A power signal (High-*γ* power) is then obtained from the squared magnitude of the Hilbert-transform of the High-*γ* band waveform, and then down-sample to 40 Hz to be in time sync with the binning of spike-counts The High-*γ* power signal is used for STRF estimation as detailed below.

### STRF estimation

#### Spike Triggered Average (STA)

A traditional STA or reverse correlation was performed on all stimulus *x*_*f*_(*t*) and response data *r*(*t*). The formal mathematical foundation for the STA used here is based on work by Krausz (1975), who extended the necessary and sufficient statistics of the input space from being Gaussian to it being Poisson. In the present case the input stimulus-state matrix *x*_*f*_(*t*) is effectively Poisson given the small *p* in the Bernoulli process. Formally, the linear kernel defines the STRF

$$STRF_{f}\left(\tau \right)=E\left[r\left(t\right)x_{f}\left(t-\tau \right)\right]$$ (4)

where *E*[⋅] is the expectation operator at each frequency and *τ* is delay with respect to the current value of time (t).

#### STRFs based on Generalized Linear Models (GLM)

GLMs have generated significant interest and progress in the study of neural encoding and decoding of perceptual and decision-making information [24, 52–56]. These advancements include the use of GLMs to estimate STRFs [23, 25, 57] based on regularized likelihood functions. STRFs computed from single or multi-unit recordings have historically been estimated using some variant of spike-triggered averaging such as reverse correlation or normalized-reverse correlation. However, these estimates tend to be noisy, and suboptimal in terms of using Gaussian assumptions rather than point or counting processes.

We considered two GLM formulations to estimate the STRF coefficients (*β*_*f*_(*τ*)) The first assumes that the spike counting process is an inhomogeneous Poisson process characterized by its conditional intensity function (CIF)

$$\lambda_{CIF}\left(t|H_{t}\right)=\text{exp}\left\{\beta_{0}+\sum_{f=1}^{N_{frequency}}\sum_{\tau =0}^{T-1}\beta_{f}\left(\tau \right)x_{f}\left(t-\tau \right)+\sum_{h=1}^{H}\alpha_{h}r\left(t-h\right)\right\}$$ (5)

where *x*_*f*_(*t*) corresponds to the gammatone-state convolution matrix. The spike count history *r*(*t*−*h*) was added to the GLM in order to factor in the contribution of past spiking activity to the expected spike-count, and *H*_*t*_ denotes the conditioning on spike history.

The second formulation used to estimate the STRF coefficients was based on the High-*γ* band response signal. The High-*γ* power signal is positive and skewed, consequently a log transformation was used to obtain a more symmetric response distribution

$$E\left[\text{logHigh}\gamma \left(t\right)|H_{t}\right]=\beta_{0}+\sum_{f=1}^{N_{frequency}}\sum_{\tau =0}^{T-1}\beta_{f}\left(\tau \right)x\left(t-\tau \right)+\sum_{h=0}^{H}\alpha_{h}r\left(t-h\right).$$ (6)

Again, *x*_*f*_(*t*) corresponds to the gammatone-state convolution matrix. The spike count history *r*(*t*−*h*) was added to the GLM to account for the contribution of current and past spiking activity to the high-gamma signal. This serves two goals. First, it partitions out the intrinsic extracellular changes in High-*γ* when a spike is observed and may include other changes in the local ensemble that might be related to spiking activity. Second, inferential statistics of the spike history contribution can be performed using standard hypothesis testing relative to the reduced models (i.e. Eqs 7 & 8)

$$\lambda_{CIF}\left(t\right)=\text{exp}\left\{\beta_{0}+\sum_{f=1}^{N_{frequency}}\sum_{\tau =0}^{T-1}\beta_{f}\left(\tau \right)x_{f}\left(t-\tau \right)\right\}$$ (7)

and

$$E\left[\text{logHigh}\gamma \left(t\right)\right]=\beta_{0}+\sum_{f=1}^{N_{frequency}}\sum_{\tau =0}^{T-1}\beta_{f}\left(\tau \right)x\left(t-\tau \right).$$ (8)

We compared the fit of the full spike counting process model (Eq 5) and corresponding reduced model (Eq 7) using the difference of deviances statistic Δ*D* = *D*_*STRF*_−*D*_STRF+spike history_, which has a sampling distribution that is well approximated by a *χ* ^2^ distribution with the degrees of freedom equal to the number of spike history covariates. An F-statistic was also computed to test the hypothesis that the full High-*γ* process model, including the spike-counts (Eq 6), differs from the corresponding reduced model without spike-count history (Eq 8), with the sampling distribution *F*(*N*_spike history_, N_observations_−N_covariates_) [58].

#### Sparse Regression with GLM

The now classic LASSO [59] method for regression models using an L1 norm regularizer ${\left\|X\right\|}_{1}=\sum_{i=1}^{n}\left|x_{i}\right|$ for covariate selection and coefficient shrinkage has generated a great deal of research and variations [26, 27, 59–64].

The L1 norm, as well as other variants, can be applied as a regularizer or penalty to both general (Gaussian) and generalized linear models. As the weighted regularizer *λ* increases, the number of zero-valued coefficients increases monotonically [60], leading to possibly only few nonzero-valued coefficients depending on the sparsity of the solution. The LASSO estimator is defined as

$$\underset{}{{\hat{\beta }}_{\lambda }=\underset{\beta \in R^{p}}{\text{argmin}}}\left(-l\left(\beta \right)+\lambda {\left\|\beta \right\|}_{1}\right)$$ (9)

where *l*(·) is the Poisson log-likelihood function of the responses *r*(*t*) and is equivalent to least-squares in the Gaussian case. The p-dimensional of estimated coefficients for a given value of *λ* is denoted ${\hat{\beta }}_{\lambda }$.

The problem in all of these cases is how to best select the weighting parameter (*λ*) of the regularizer. In most cases *λ* is chosen by criteria aimed at maximizing prediction accuracy under constraints. These are generally either Akaike or Bayesian information criteria based, or through some form of cross-validation. We are primarily interested in selecting STRF coefficients based on null hypothesis rejection, rather than optimizing its prediction accuracy. One alternative approach to criteria-based or cross-validation based selection of *λ* is a so-called discovery-based selection using multiple permutations of the response variable [65–68]. The idea is to select a conservative model that would tend to include no covariates in the model under permutation of the response; that is, when the association between covariates and responses have been destroyed by the random permutation process.

**Fig 4:**
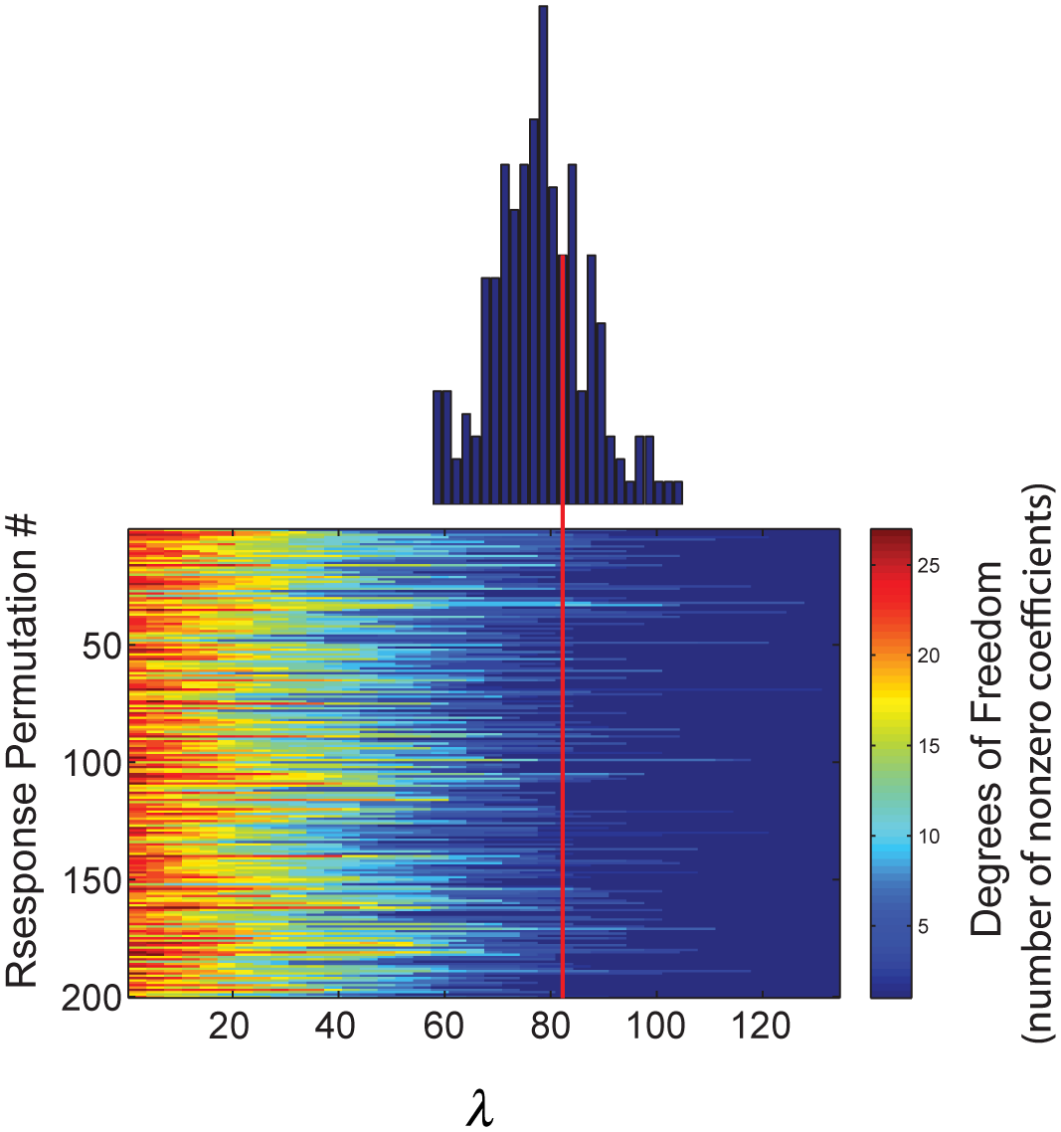
Discovery-based selection of lambda (*λ*). Discovery-selection by permutation of the response *r*(*t*) to identify the minimum lambda necessary to reduce the degrees of freedom to zero. Empirical distribution shows the histogram of minimum *λ* over 200 repeated permutations and serves as the null distribution. The red arrow denotes the median of the distribution that we designate as the optimal *λ*.

The permutation process was performed as follows. The electrophysiological responses were shuffled and the covariates ${\hat{\beta }}_{\lambda }$ are estimated using Eqs 5 and 6 for a range of regularizer weights *λ* that insures that at some point along this interval all ${\hat{\beta }}_{\lambda }$ s zero out. The number of degrees-of-freedom corresponds to the number of non-zero coefficients in the GLM solution [62]. So, zeroing out all coefficients represents zero degrees-of-freedom. In cases when an intercept is included in the model it is spared any shrinkage, and would therefore always have 1 degrees-of-freedom. The value representing the smallest regularizer that zeros out all of the free covariates is then recorded. This process is repeated with a large number of repetitions to generate an empirical null distribution of minimum *λ*. Fig 4 shows an example of 200 repetitions of the GLM solution, degrees-of-freedom as a function of *λ* values. The degrees-of-freedom always declined monotonically as *λ* increases until the minimum *λ* (0 or 1) degrees-of-freedom is reached. The distribution of minimum *λ* s is shown above the repetition matrix with the median value indicating a *λ* value of approximately 80. This so-called optimal value of *λ* would then be selected for estimating the betas for the true electrophysiological response. In contrast to the cross-validation method, the discovery-based method is rooted in bootstrapped inferential statistics with advantages that preserve family-wise error rates. The intuition is that the selected lambda reflects the expectation of the null distribution for a chance only STRF structure. Observed structure having non-zero coefficients would be unexpected under the null permuted distribution.

#### Group Lasso GLM

Although the GLM is constructed assuming independence between frequency channels, this in fact is not the case given how frequency is mapped onto the mammalian cochlea. Dependencies exist across both cochleotopic mapped frequencies as well as time. To address this condition, frequency-time local neighborhoods or groups were constructed to inform the regularized GLM to treat frequency-time groups as possibly sparse while preserving the internal structure of regions of the STRF. We constructed a 50 (frequencies) by 40 (time-bins) element grid with 4 x 4 element labeled groups for a total of 130 groups, as well as a single group assignment of the spike-count history. A 4x4 patch would represent about a 4 mm linear interval on a 35 mm human cochlea and 100 ms interval of time. The solution for the regularized GLM with spike count history included one additional group label for these covariates.

We used a variant of sparse regression methods known as the Group Lasso[27]. The estimator is defined as

$$\underset{}{{\hat{\beta }}_{\lambda }=\underset{\beta \in R^{p}}{\text{argmin}}}\left(-l\left(\beta \right)+\lambda \sum_{g=1}^{G}{\left\|\beta_{g}\right\|}_{2}\right)$$ (10)

where again *l*(·)is the Poisson log-likelihood function and is related to least-squares in the Gaussian case. The group-wise L2 norm ${\left\|X\right\|}_{2}={\left(\sum_{i=1}^{n}\left|x_{i}^{2}\right|\right)}^{1/2}$ in Eq 10 is a form intermediate between and L1 and L2 norm. It encourages that either all of the group members of *β*_*g*_ are equal to 0 or none are. The L2 norm encourages some shrinkage within the group *β*_*g*_, but they will not shrink to 0.

The Group Lasso solutions were obtained using interative shrinkage-thresholding algorithms (ISTA). The estimates of the STRF *β*_*λ*_ was based on the implementation of proximal (ISTA) methods in a Matlab toolbox SPAMS: SPArse Modeling Software [69]. These methods solve linear inverse problems, which are an extension to the classical gradient algorithm [70]. They can solve a large class of sparse approximation problems with different combinations of loss and regularizations, and therefore represent unified methods for solving L1, Group L1/L2 penalized GLMs, both Poisson and Log-Normal.

## Results and Discussion

### Spike-count STRF from STA and GLM with Independent (L1) and Group (L1/L2) penalties

**Fig 5:**
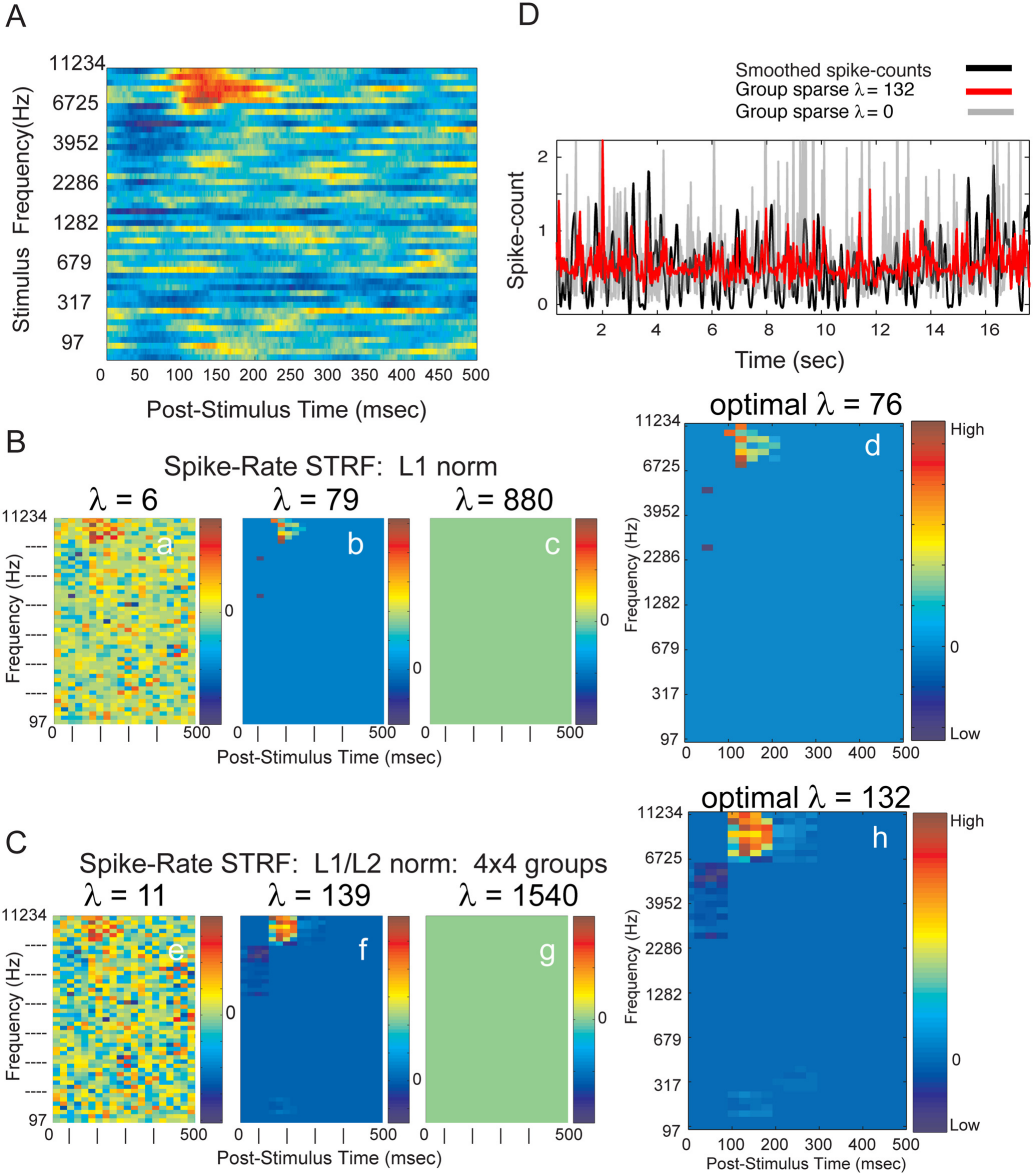
STRF estimated from STA and from GLM. **(A**) STRF from STA. Stimulus center frequencies ranged from 97 to 11,234 Hz. Spike-times were binned at 1 ms resolution. (**B**) Evolution of Spike-count STRF from GLM as a function of increasing *λ* for L1 norm LASSO: (**a**) very low values of *λ* lead to noisy estimates, (**c**) at very high values of *λ* all covariates are zero valued, (**d**) at optimal value of *λ* chosen from discovery-based selection. (**C**) Evolution of Spike-count STRF from GLM as a function of increasing *λ* for L1/L2 norm group LASSO: (**e**) very low, (**g**) very high, and (**h**) optimal values of *λ*. Optimization selects or removes, simultaneously, all the covariates forming a group. Groups are composed of 4x4, adjacent and non-overlapping covariates. **(D)** Predicted discharge *λ*_*CIF*_(*t*|*H*_*t*_) from representative segment of gammatone stimulus, with and without sparse-group regularization. Group-sparse regularized GLM consistently improved the prediction of validation data over non-regularized GLM prediction of expected spike-counts. Correlation coefficients are 0.133 with regularization (red) and 0.066 without regularization (gray). Neural responses from S178, electrode contact #4.

A traditional STA (reverse correlation) was applied to the input gammatone stimulus-state matrix and output spike counts from Heschl’s gyrus recordings to estimate STRFs. The Poisson discrete stimulus-matrix sidesteps the need for STA normalization [71]. A representative example of an STRF derived from reverse-correlation is shown in Fig 5. for one representative electrode contact. The STRF structure is well-defined in frequency-time with evidence of suppression that precedes an increase in discharge rate.

We examined the effects of titrating the penalizing weight *λ* from low values to high values on Spike-count STRFs. As the sparsity inducing norm was weighted more heavily, a greater number of covariates are zeroed out(i.e. removed) from the solution. This occurred universally regardless of the type of norm, L1 or L1/L2, on the spike-count response variable. This can be observed in Fig 5B and 5C as a progressively less noisy STRF emerges as *λ* increases the effect of the penalties. At high values of *λ* the coefficients are completely zeroed out. The right panel shows the STRF at the optimal *λ* selected by the permutation method.

The L1 norm assumes that every cell in the STRF is independent and therefore targeted uniformly for deletion. By comparison, the L1/L2 norm targets local group structure for deletion or retention. In either case, the permutation method of *λ* selection identifies an STRF structure that appears as a less noisy representation compared to that of the reverse correlation STRF shown in Fig 5A.

The predicted spike-count was computed on validation data following estimation of the STRF using the Poisson-GLM (Eq 5) on a training data set (Fig 5D). The recorded spike-counts were smoothed forward and backwards using a second-order lowpass Butterworth filter with a cutoff of 6 Hz. Predicted spike-counts were generated with (*λ* = 132) and without (*λ* = 0) sparse-group regularization, demonstrating the predictive advantage of the sparse-group GLM approach over classical GLM regression.

#### Independent (L1) and Group (L1/L2) penalties on High-*γ* power STRF

High-*γ* power (70 to 150 Hz) has been observed in intracranial LFPs and ECoGs in the human brain under a number of experimental conditions. The relationship between High-*γ* power and spiking neural activity is complex, and represents an active area of study[28, 32, 72, 73]. A wide range of spike-gamma coupling has been observed in human auditory cortex, primarily reflecting the degree of correlation between adjacent neurons [74]. A number of recent studies have shown a tight coupling of High-*γ* power and spiking activity, suggesting that High-*γ* may reflect a neural correlate of population firing rate [30, 72, 75, 76].

In primary auditory cortex of the non-human primate, there is good correspondence between the pure-tone spectral sensitivity of multiunit activity from middle cortical layers and higher gamma frequency band activity in more superficial laminae [72]. In contrast, lower frequency bands of the LFP had the poorest correlation with multiunit pure-tone tuning. Whether this organizational scheme is applicable to non-core auditory fields, like those on the lateral surfaces of non-human primates and humans, is largely unknown and yet to be determined. In our present studies, it has not been possible to obtain either multiunit or cluster data from locations on the lateral surfaces and, therefore, we cannot directly address this issue. High-*γ* and spiking activity have been shown to be highly correlated in area V1 of the macaque when presented with a movie with both signals tuned to visual features. High-*γ* and spiking activity have been shown to be highly correlated in area V1 of the macaque when presented with a movie with both signals tuned to visual features. High-*γ* power signal also carried the maximal visual information about the movie [77]. These results are consistent with the view that High-*γ* and spiking activity might arise within the same localized cortical ensemble, with feedback of spiking pyramidal neurons providing one source of the High-*γ* power. This would be consistent with the idea that spiking activity reflects the output of large pyramidal neurons, and LFPs representing area input, such as synaptic potentials, and slower local cooperative activity [78]. We have previously shown that event-related High-*γ* power reflects a functional organization of latencies in human auditory cortex [33] and spectral organization [34].

**Fig 6:**
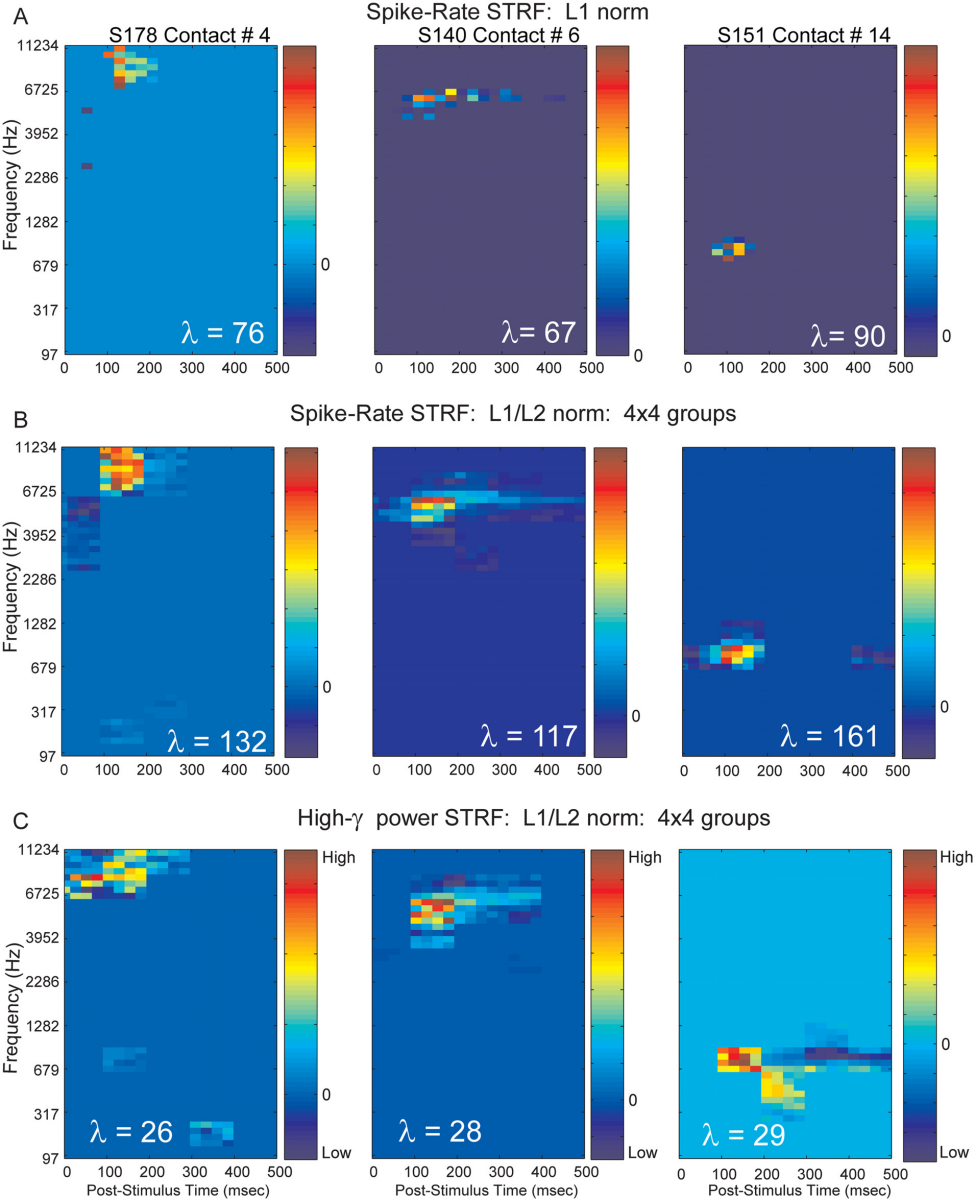
Spike-count and High-*γ* power STRFs derived with sparse GLM models. Neural responses from S178 electrode contact #4 used in left column, responses from S140 electrode contact #6 used in middle column and from S151 electrode contact #14 used in right column (See also Fig 1). Optimal *λ* values are shown on insets. (**A**) Spike-count STRF using L1 sparsity-inducing norm. (**B**) Spike-count STRF using L1/L2 norm regularization that exploits group structure when covariates are partitioned into neighborhoods, or groups. In this case, optimization selects or removes all the variables forming a group. Groups are composed of 4x4, adjacent and non-overlapping covariates. (**C**) High-*γ* (70 to 150 Hz) band power STRF from L1/L2 norm regularization that exploits group structure. Groups are composed of 4x4, adjacent and non-overlapping covariates.

STRFs derived from low frequency band (2 to 40 Hz) LFPs are much more broadly tuned than spike based auditory cortex STRFs in the anesthetized cat [47], and similarly in primary auditory cortex of the guinea pig [79]. However we observed STRFs based on High-*γ* power to be comparable in both frequency and temporal tuning to STRFs based on spiking activity. STRFs based on spike responses and High-*γ* power are shown for 3 electrode contacts recorded in Heschl’s gyrus of 3 subjects. STRF differences between the 3 rows correspond to the L1 norm spike-count STRF, the L1/L2 group spike-count STRF, and the L1/L2 group High-*γ* power STRF. The strength of applying the grouped penalized GLM (Fig 6B) compared to the individual covariate removal by the L1 regularizer (Fig 6A) is evident in the preservation of structure internal to the STRF. High-*γ* power STRFs based on a grouped regularizer are shown in Fig 6C. Although some differences between spike based and High-*γ* emerge, such as a lack of the identified early suppressed area evident in the spike-count STRFs for contact #4, they are remarkably similar in terms of their locations and tuning in frequency and time. Contact #14 was striking in that, although the tuning bandwidth is similar between spike-count and High-*γ* power, a complex pattern of enhancement and suppression was revealed; suggestive of a spectral motion detector for the STRF based on High-*γ* power.

#### Contributions of spike-count history to spike-count and High-*γ* power STRF estimation

Given the strong evidence that High-*γ* power covaries with spiking activity [28–31], we investigated the degree to which spiking activity covaries with High-*γ* power when driven by our gammatone stimuli. The STRFs based on these two different signals are similar in structure and extent, suggesting a similar underlying mechanism. The GLM provides a method for partitioning out the covariance of the extrinsic input stimulus from that of the intrinsic contributions of the spiking activity, and it’s history.

**Fig 7:**
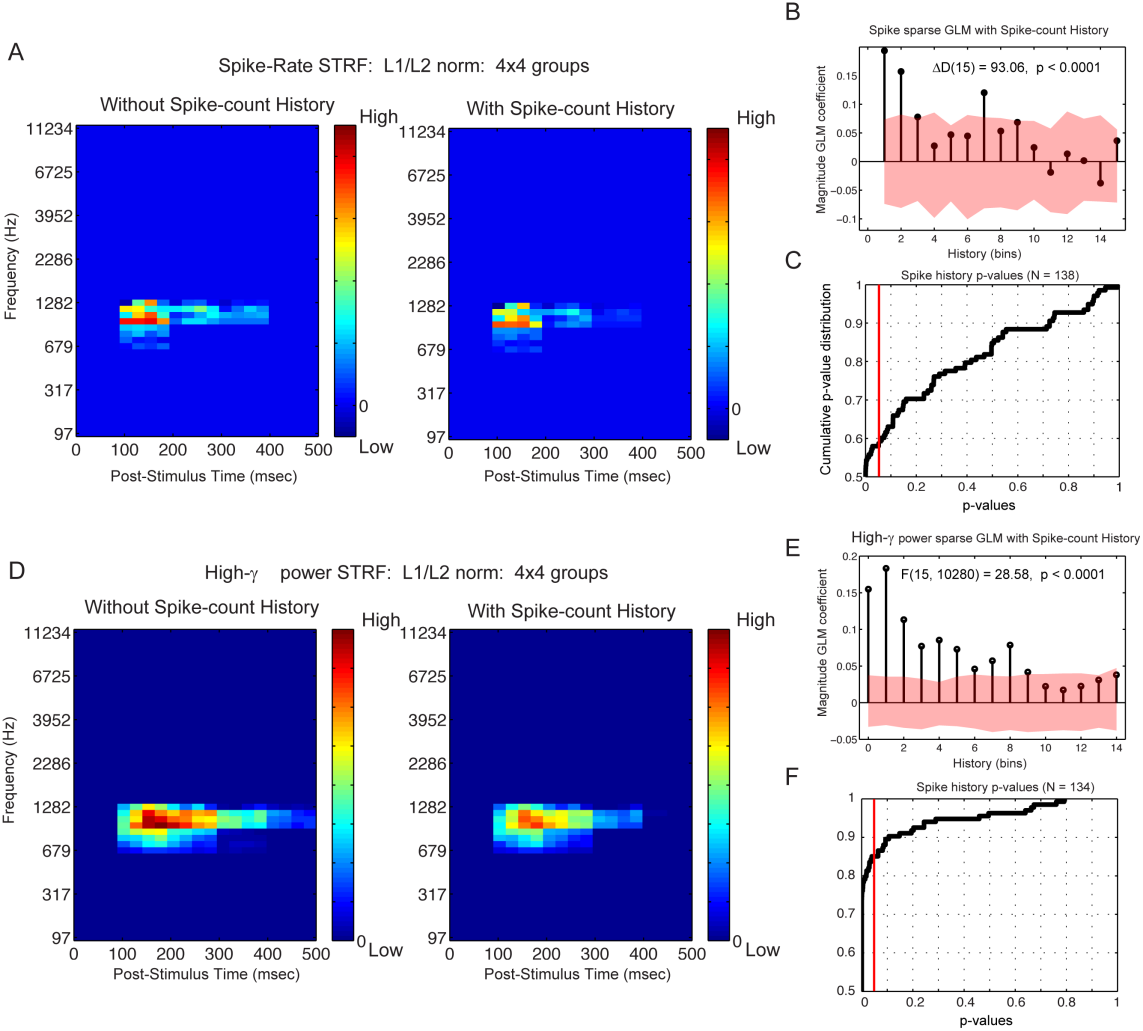
Spike-Count History contribution to spike-count and High-*γ* band power STRFs. Neural responses from S178, electrode contact #11. Anatomical location shown in Fig 1. (**A**) Spike-count STRF estimated with L1/L2 sparsity-inducing norm and group structure GLM with Poisson distribution to link responses to predictors, without and with 350 ms spike-count history. (**B**) Magnitude of GLM spike-count history coefficients decreases with increasing history (i.e. time elapsed since current spike-count prediction). Shading represents 95% central range of null distribution estimated from permuted random shuffling of responses. (**C**) Cumulative distribution of p-values testing the contribution of spike-count history to current spike-count activity driven by gammatone stimuli. All p-values were adjusted for false discovery rate. (**D**) High-*γ* power STRF estimated with L1/L2 sparsity-inducing norm and group structure GLM with Poisson distribution to link responses to predictors, without and with 350 ms spike-count history. (**E**) Magnitude of GLM coefficients decreases with increasing spike-count history (i.e. time elapsed since current High-*γ* band power prediction). (**F**) Cumulative distribution of p-values testing the contribution of spike-count history to current High-*γ* power activity driven by gammatone stimuli. All p-values were adjusted for false discovery rate.

The group sparse STRFs derived from spike-count alone is shown in Fig 7A and 7B. The overall structure, latency and center frequency are again similar to one another. Notably, the internal structure of the High-*γ* power STRF is graded and somewhat more sustained compared to the spike-count STRF. We were interested in the contribution of the simultaneous spike-count, recorded from the same electrode, as well the contribution of the spike-count history. We used the group sparse GLM with added covariates for the spike-count history, which were treated as a single, unique group. The covariate coefficients are shown in Fig 7B and 7E, where the index corresponds to time bins advanced back in time (350 ms). The permuted spike counts included in the GLM were replicated 100 times, and the 95% central range of the empirical null distribution are shown with an overlaid red ribbon.

The contribution of past spike-counts to both the driven spike-counts and High-*γ*power signal were reliable back to about 200 ms. As expected, the spike-count covariate weights trended downward back in time. The difference in deviance statistic Δ*D* and F-statistic were computed to test the hypothesis that the full models, including the spike-counts (Eqs 5 and 6), differs from the reduced models without spike-count history (Eqs 7 and 8); Δ*D* (15) = 93.06, p(false discovery rate) < 0.0001 for the driven spike-counts and F(15, 10280) = 28.58, p(false discovery rate) < 0.0001 for the High-*γ* power signal [58]. In 60 percent of the 132 spike-count recordings and in 85 percent of the 134 High-*γ* power recordings, introducing the spike-count history significantly improved the model (Fig 7C and 7D). This feedback of spiking activity built into the GLM could reflect the local spiking ensemble behavior influencing the LFP dynamics [31]. Notably, both STRFs become somewhat more localized in time when the spike-count history is regressed out. The time lag was also consistent with LFP and spike observations in non-human primate primary visual cortex in response to semi-natural movies [28].

#### Relevance to human HG frequency tuning

We are aware of only two published studies that examined single- or multi-unit frequency tuning in human auditory cortex [12, 13]. Coupling between intracranial LFP and spiking activity in human auditory cortex has been reported in response to an audio-visual movie [74]. The selectivity to naturalistic stimuli was described as complex with both broad and narrow tuning, however a detailed analysis of frequency tuning was not performed. Our analysis of Heschl’s gyrus electrophysiology also supports similar complexity in spectrotemporal tuning. Rather than random tones or naturalistic sounds, we used gammatones that were uniformly mapped onto the human cochlea. Using advanced statistical modeling with sparse constraints we have revealed STRFs that are less noisy than that which could be estimated from traditional reverse-correlation. Furthermore, the GLM formulation allows a natural introduction of both intrinsic (spiking activity) and extrinsic (auditory) stimulation.

Unlike Bitterman et al. [12] we generally observed both broad and relatively narrowly tuned STRFs based on a similar uniformly tiled spectrotemporal stimuli. Our stimuli were presented as a random Bernoulli sequence, which by chance in any 50 ms interval in time, could contain between zero and six simultaneous gammatones. It is perhaps the case that our acoustic stimulus had characteristics that resembled natural acoustics to a greater degree than that of the Bitterman at al. synthetic stimuli. Complex patterns were observed in both spike-count and High-*γ* power based STRFs (Fig 6). Contact #14 (Fig 6C, right panel) was localized to the anterolateral Heschl’s gyrus, and can be interpreted as a non-core region of human auditory cortex [33]. High-*γ* power in this area may provide a window into higher-order network activity, which was not captured by spike-counts.

Although their sample size was small (47 measurements), Bitterman and colleagues reported that about 87% of their units exhibited narrow, well-circumscribed response areas, and that fully 60% responded reliably to only a single frequency out of the 6 or 18 tested tones. The average bandwidth was a remarkable 0.08 octave with best frequencies ranging from 250 to 2 kHz. Their results argue for a view of human cortical frequency tuning that is substantially narrower than that typically reported for the human periphery (0.16 octave) and auditory cortex in animal models. By contrast, our estimates of octave bandwidth (minimum = 0.21, maximum = 2.26, median = 0.25) from clusters is more in-line with those from human periphery (0.27 octave) and non-human mammals (0.3 to 1 octave). Our sample size in comparable to that of Bitterman, however, estimated best frequencies in Hz (minimum = 154, maximum = 9770, median = 2576) do indicate that different populations were sampled in the two studies.

#### Relationship of temporal processing derived auditory fields to STRF frequency tuning

**Fig 8:**
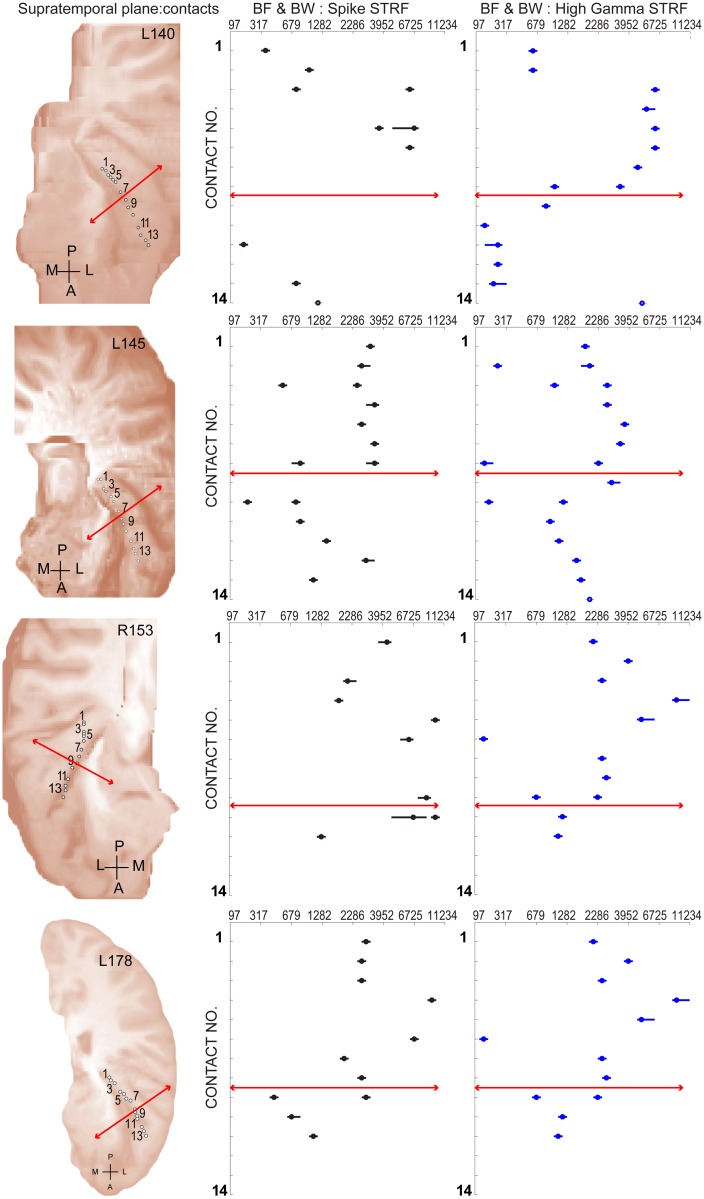
Best frequency and bandwidth estimated for sites within Heschl’s gyrus. **(Left column**) Fourteen recording locations in each subject were projected to the surface and marked with open circles. Solid red line marks the transition from core to non-core fields estimated with click train stimuli. (**Middle column**) Open circles mark a single or multiple best-frequency (BF) value for each location estimated from spike-count STRF. Bandwidth (BW) is depicted by a solid black line centered on the best-frequency for each location. (**Right column**) BF and BW mapped using High-*γ* power STRF.

This study analyzed the responses from Heschl’s gyrus localized electrode sites obtained from 5 subjects. A single hybrid depth electrode containing 14 microwire contacts was implanted in each subject from which recordings could yield spikes and local field potentials. Here we illustrate the locations of recording sites along the length of a depth electrode within Heschl’s gyrus, and provide estimates of best-frequencies and spectral bandwidth that were obtained from their corresponding STRF. A previous report [8] from our laboratory focused attention on the temporal processing of repetitive acoustic transients by auditory core and non-core fields of Heschl’s gyrus. In that study, the amplitude of the average evoked potential (AEP) elicited by click-trains declined from a maximum value at sites located in posteromedial Heschl gyrus to a minimum value for sites located successively more anterolaterally. Together with a correlated decrease in the frequency-following component of the AEP, this spatial response pattern was used as evidence for a transition from core to lateral belt (i.e. non-core) auditory fields. We employed the method of Brugge and colleagues [8] to locate this laterally located transition region. The mapping for 3 of these cases (L140, L145, R153) are also reported in their previous study (see their Fig 3). One subject’s mapping (R151) is excluded from both reports since many of the recording sites in that case were localized outside of Heschl’s gyrus. Fig 8 illustrates the resulting transition location (solid red line) for these 4 subjects as depicted within the posteromedial-to-anterolateral recording locations along the long axis of the Heschl’s gyrus. The trajectory of the electrode and locations of microwire contacts in each subject are shown (1st column) projected to the surface of the supratemporal plane. This spatial mapping layout was also used to examine the distribution of best-frequency (BF) and spectral band-width (BW) as estimated from the STRF obtained at that location using both spike clusters (2^nd^ column) or High-*γ* (3^rd^ column) response metrics. A Pearson product-moment correlation coefficient was computed to assess the relationship between BF as estimated from the STRF obtained using spike clusters and High-*γ* response metrics. In each of these 4 subjects there was a positive correlation between the two variables (L140: r = 0.88, n = 8, p = 0.003; L145: r = 0.79, n = 15, p = 0.0003; R153: r = 0.84, n = 9, p = 0.004; L178: r = 0.84, n = 11, p = 0.0002).

For all subjects, the highest BFs occupied locations in the posteromedial half of the sampled area. Furthermore, BF and BW typically demonstrated a change in values at or near the lateral transition region (solid red line) derived from click-rates. The lateral transition region changes in BF were often marked by an abrupt decrease in value and/or evidence of two separate BW spectral ranges where the additional range occupies the lower value. Brugge and colleagues [8] found no evidence from temporal processing to support a 2nd transition region located at an extreme posteromedial position in Heschl’s gyrus. In 3 of the current subjects (L140, R153, L178), BF values from recordings at this extreme (e.g. contacts, 1, 2, or 3) are seen to be lower than those at the next more anterolateral contacts. One interpretation of this pattern is that the tonotopic gradient reverses at the location of highest BF.

In reviewing results from non-human primates and other animals, Schönwiesner and colleagues [80] pointed out that borders between core fields are often marked by such reversals, whereas borders between core and belt fields are often not associated with a reversal in the tonotopic gradient. In this regard, the abrupt change in BF demonstrated here near the lateral transition region may reflect the latter situation and signal a transition to a non-core field. A recent synthesis of findings from human fMRI, myeloarchitectonic [80], and other functional criteria suggest that a single best-frequency gradient within a relatively small, circumscribed region around the middle part of Heschl's gyrus represents the only coherent tonotopic gradient; presumably that of the primary auditory cortex [80]. Together with our findings from the temporal processing of acoustic transients, these results provide support for the interpretation of one core field with a low-to-high progression of BF that corresponds to a posteromedial to anterolateral progression of recording sites.

There are several limitations of the group sparse GLM approach. First, the use of non-overlapping all-or-none groups generates STRF patterns that can appear truncated. The first step towards addressing this limitation is the construction of over-lapping groups, that could better incorporate biological properties [5, 8, 9]. This would have the effect of reducing the grouped edges, while preserving the emergent complex and multi-modal structure of the STRF. Second, would be to apply the group sparse STRF method to natural auditory stimuli, such as speech. The filtering of continuous speech with the derived bank of gammatone impulse responses may provide important new insights into cortical processing not revealed with less naturalistic stimulus sets. This would come at the cost of abandoning the known, structured randomness of the stimulus, but unlike traditional reverse-correlation is not necessary when using the GLM approach [23, 81].
